# Insights into spatial configuration of a galactosylated epitope required to trigger arthritogenic T-cell receptors specific for the sugar moiety

**DOI:** 10.1186/ar2291

**Published:** 2007-09-11

**Authors:** Simon Glatigny, Marie-Agnès Blaton, Julien Marin, Sylvie Mistou, Jean-Paul Briand, Gilles Guichard, Catherine Fournier, Gilles Chiocchia

**Affiliations:** 1 Institut Cochin, Université Paris Descartes CNRS (UMR 8104), 27 rue du Fbg Saint Jacques, Paris, F-75014, France; 2 INSERM U567, Département d'Immunologie, 27 rue du Fbg Saint Jacques, Paris, F-75014, France; 3 UPR 9021 CNRS – Immunologie et Chimie Thérapeutiques (ICT), Institut de Biologie Moléculaire et Cellulaire, 15 rue René Descartes, 67084 Strasbourg Cedex, France

## Abstract

The immunodominant epitope of bovine type II collagen (CII256–270) in A^q ^mice carries a hydroxylysine-264 linked galactose (Gal-Hyl^264^), the recognition of which is central to the development of collagen-induced arthritis. This study explores the molecular interactions involved in the engagement of T-cell receptors (TCRs) with such epitopes. Responses of three anti-CII T-cell hybridomas and clone A9.2 (all sharing close TCR sequences) to a panel of CII256–270 analogues incorporating Gal-Hyl^264 ^with a modified side chain were determined. Recognition of naturally occurring CII256–270 peptides by either group of T cells depended strictly upon the presence of the carbohydrate and, more precisely, its intact HO-4 group. Modifications of primary amino group on the hydroxylysine side chain eliminated T-cell reactivity, notwithstanding the presence of the galactosyl moiety. Moderate stereochemical changes, such as altered sugar orientation and methylation at the galactose anchor position, were still permissive. Conversely, robust transformations affecting the relative positions of the key elements were detrimental to TCR recognition. To conclude, these data provide strong new experimental evidence that integrity of both galactose HO-4 and hydroxylysine side chain primary amino groups are mandatory for activation of anti-Gal-Hyl^264 ^TCRs. They also indicate that there is a certain degree of TCR plasticity in peptide-TCR interactions.

## Introduction

Rheumatoid arthritis (RA) is a prevalent autoimmune disease that is characterized by synovial inflammation and pannus formation, which lead to irreversible cartilage and bone degradation. Although many candidate autoantigens have been suspected of initiating a deleterious immune response in RA, none of them have been formally identified as such. There is considerable evidence in the literature implicating post-translational modifications of proteins in the pathophysiological processes of human autoimmune disorders via creation of new antigenic epitopes [1,2]. More recently, work from various groups outlined the possible contributions made by citrullination of arginine residues in a number of different proteins to the breakdown of self-tolerance in RA and its influence of disease severity [3,4]. Type II collagen (CII) is another probable target autoantigen that may be involved in the pathogenesis of RA. This is supported by detection of CII-specific antibodies in the serum of patients and the isolation of T cells reactive to CII from affected synovial tissues [5]. In addition, a RA-like disease can be induced in susceptible strains of rodents and nonhuman primates upon immunization with CII [6,7].

Native CII is a fibrillar protein composed of three identical α1(II) chains derived through extracellular processing of pro-collagen. Its synthesis involves a number of post-translational modifications, including hydroxylation of the majority of prolines and lysines that are located in the Y position of the Gly-X-Y repeating triplet. Furthermore, during biosynthesis of cartilage pro-collagen, more than two-thirds of hydroxylysine residues undergo glycosylation, which consists of covalent linkage of the monosaccharide galactose (Gal-Hyl) or the disaccharide glucosylgalactose [8]. During the past decade, a number of studies conducted in H-2^q ^mice converged to demonstrate that the high carbohydrate content of CII is associated with its arthritogenicity [9,10]. Studies have also documented the crucial role played the glycosylation carried by the CII256–270 epitope (the immunodominant epitope of bovine CII) in triggering the immune T-cell response after priming with heterologous native CII in complete Freund's adjuvant (CFA) [11,12]. Interestingly, predominant immunogenicity of this glycosylated epitope was also identified both in humanized transgenic mice lacking endogenous major histocompatibility complex (MHC) class II molecules but expressing RA-associated human leucocyte antigen-DR4 and in severely affected RA patients [13].

A few years ago, while investigating pathogenic T-cell responses in DBA/1 (H-2^q^) mice suffering from collagen-induced arthritis (CIA), we isolated a recurrent T-helper-1 clone, named A9.2, expressing a T-cell receptor (TCR)αβ that shares almost identical complementarity-determining region (CDR)3αβ with those carried by three CII-specific CD4^+ ^hybridomas generated previously [14,15]. Not only were these cells consistently identified in lymph nodes from CII-primed mice, but also they were shown to modulate clinical symptoms of CIA in adoptive transfer experiments [15] or using T-cell vaccination protocols [14,16,17]. Such regulatory effects suggests that these T cells play a key role as effectors in the pathogenic process of CIA, rendering them appropriate targets for peptide therapy in this disease. In the present study we evaluated the molecular interactions involved in the recognition of a glycosylated epitope by TCRs of cells that drives CIA.

## Materials and methods

### Synthetic peptides

**Figure 1:**
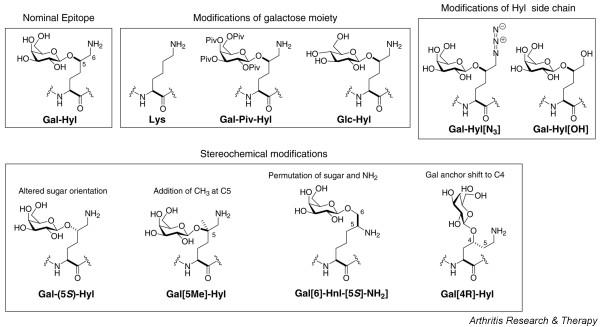
Immunodominant CII256–270 peptide analogs. Shown is a schematic representation of the immunodominant epitope of bovine type II collagen (CII256–270) peptide analogues synthetized in this study [20,21].

The sequences of bovine and mouse CII(256–270) with and without post-translational modifications at Pro^258 ^and Lys^264 ^are the following: Gly^256^-Glu-(Pro/Hyp)-Gly-Ile-Ala-Gly-Phe-(Lys/Gal-Hyl)-Gly-Glu-Gln-Gly-Pro-Lys^270 ^(bovine) and Gly^256^-Glu-(Pro/Hyp)-Gly-Ile-Ala-Gly-Phe-(Lys/Gal-Hyl)-Gly-Asp-Gln-Gly-Pro-Lys^270 ^(mouse). The panel of modifications incorporated at the Gal-Hyl^264 ^side chain in the sequence of the bovine or mouse immunodominant CII(256–270) glycopeptide is shown in Figure 1. The synthesis of *N*-Fmoc-protected Gal-Hyl residue and glycosylated building blocks with modifications at the Gal-Hyl side chain (specifically, GalPiv-Hyl, Gln-Hyl, Gal-Hyl[N_3_], Gal-Hyl [OH], Gal [5*S*]-Hyl and Gal [5Me]-Hyl and Gal[6]-Hnl-[5S]-NH_2_), as well as corresponding glycopeptides, were previously reported in detail [18,19]. The synthesis of the Gal [4R]-Hyl building block and corresponding glycopeptide will be described elsewhere.

### T cells and hybridomas

Three anti-CII CD4^+ ^T-cell hybridomas (A2G10, A8E2 and A9E5) were used in the present study. They were derived by fusion of lymph node cells from CII-primed DBA/1 (H-2^q^) mice and the BW5147 thymoma (mutant TCRαβ^-^) [14]. The three anti-CII CD4^+ ^T-cell hybridomas used were derived from different mice and thus represent individual clones.

The anti-CII T-cell clone A9.2 was isolated from the lymph nodes of CII-immunized DBA/1 mice [15]. All of the cells were cultured in RPMI 1640 glutamax supplemented with antibiotics, 5 × 10^-5 ^mol/l mercaptoethanol, 10 mmol/l HEPES, 2 mmol/l sodium pyruvate (GIBCO, Burlington, ON, Canada) and 7% heat-inactivated foetal calf serum, referred to below as 'complete medium'.

### Determination of *in vitro *T-cell clone and hybridoma reactivity

T-cell responses were assessed by means of proliferation measurement for A9.2 clone and quantification of IL-2 secretion for T hybridomas. Antigen-presenting cells (APCs) used were either DBA/1 irradiated spleen cells (5 × 10^5 ^cells/well) or paraformaldehyde-fixed M12.C10 cells (10^5 ^cells/well), and an I-A^q+ ^B lymphoma that we generated [20]. In all of the tests, T-cell clone (3 × 10^4 ^cells/well) or T hybridomas (10^5 ^cells/well) were co-cultured in triplicates with APCs in the presence of increasing concentrations of glycopeptides in a total volume of 200 μl of complete medium. The A9.2 cell cultures were incubated at 37°C in 5% carbon dioxide for 3 days. [^3^H]thymidine (0.5 μCi/well) was added during the last 16 hours of culture, and radioactivity incorporated by the cells was determined by liquid scintillation counting. This clone possessed a T-helper-1 phenotype, based on its high secretion of interferon-γ but not of IL-4 or IL-5 in response to stimulation with antigen. The interferon-γ production parallels the proliferation for all modified peptides tested. Regarding the T hybridoma cultures, supernatants were collected after 24 hours of incubation and frozen at -20°C. Thawed supernatants were tested for their ability to support CTLL-2 (Cytotoxic T cell line IL-2 dependant) proliferation following the procedure of [^3^H]thymidine incoporation described above. The results were expressed as mean of triplicates after deduction of mean background obtained by co-culture of T cells and APCs without peptide (Δ counts/min) or as stimulation index (ratio of peptide-stimulated to medium-treated co-cultures).

The studies were approved by the Cochin institute committee on animal care. The agreement reference number to conduct experiments in living animals is 75–777, and the animal facility agreement reference number is 3991.

### Assay for evaluation of *ex vivo *T-cell responses

Depending on the experiment being conducted, cell suspensions were prepared from one of two sources. The first is afferent lymph nodes, collected 11 days after foot pad immunization with 100 μg peptide in CFA. The second is peripheral lymph nodes and spleen of mice immunized with 100 μg CII in CFA and challenged with the same dose of CII in incomplete Freund's adjuvant. One week later, single cell suspensions were prepared and enriched in CD4^+ ^lymphocytes using the SpinSep™ kit (StemCell methodologies inc, Vancouver, Canada) following manufacturer's recommendations. In both the cases, cells were stimulated for 4 days (in the presence of APC when responder cells were CD4^+ ^lymphocytes) with increasing concentrations of peptides. Cell proliferation was measured by [^3^H] thymidine incorporation as described above.

### Inhibition experiments

When inhibition experiments were performed, various quantities of inhibitory peptides were pre-incubated with APCs 2 hours before the stimulatory peptide was added, at the indicated concentrations, together with the T-cell hybridomas. After 24 hours, 100 μl of the supernatant was transferred to a new plate, which was subsequently frozen to kill any transferred T-cell hybridomas. The reactivity of the T-cell hybridomas was tested with a CTLL assay as described above. All tests were conducted in triplicate.

## Results

### Recurrent T-cell clones in CIA recognize exclusively post-translational modifications of CII

**Figure 2:**
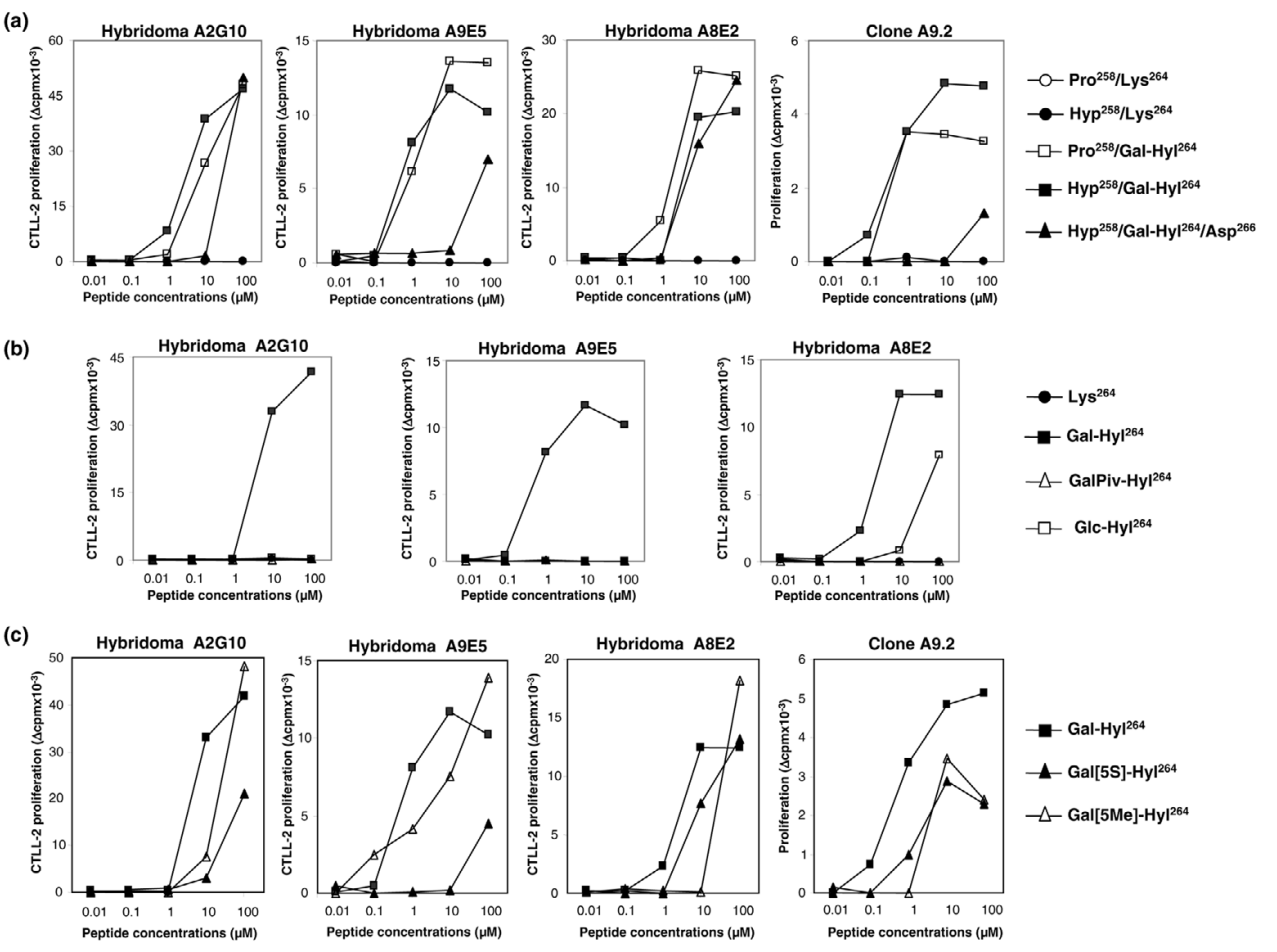
T-cell reactivities of hybridomas A2G10, A9E5 and A8E2, and clone A9.2 to several CII256–270 analogues. T cells were stimulated with increasing concentrations of synthetic peptides in the presence of antigen-presenting cells, and their responses were assessed by quantification of interleukin-2 secretion in the supernatant or measurement of proliferation for the hybridomas and the T-cell clone, respectively. Data are expressed as means of two to five individual experiments. **(a) **Recognition of a panel of naturally occurring peptides synthesized with or without the potential post-translational modifications at positions 258 and/or 264. The murine peptide comprises a Glu^266^→Asp substitution. **(b) **Loss of hybridoma reactivity following changes targeting the galactose molecule linked to Hyl^264^. **(c) **Comparison of T-cell hybridoma and clone reactivities to cognate glycopeptide and derivatives modified at sugar anchor position. CII256–270, immunodominant epitope of bovine type II collagen.

Three T-cell hybridomas (named A2G10, A8E2 and A9E5) and one T-cell clone (named A9.2) specific for CII were previously generated from bovine CII primed mice. All of these cells, which express closely related TCRs, were found to react with the arthritogenic CB11(II) fragment purified from native CII but not with any of the overlapping synthetic peptides (20 mer) that mimic the CB11 sequence, even when prolines (at Y position of Glu-X-Y triplets) were hydroxylated (not shown). It is likely that these cells failed to respond to deglycosylated CII, thus suggesting that they all recognize a carbohydrate carrying epitope. Sequential enzymatic cleavages of natural CB11 peptide allowed us to assign the reactivity to a fragment comprising the immunodominant CII256–270 epitope, in which the hydroxylation and subsequent galactosylation of Lys^264 ^was shown to be crucial for stimulation of some T hybridomas [12]. The strong dose-dependent activation of A9.2 clone and the three T hybridomas with the synthetic Gal-Hyl^264 ^glycopeptides and the lack of reactivity against the same unmodified Lys^264 ^peptides, even at high concentrations, validated this assumption (Figure 2a).

To investigate the fine specificity of the T cells and to determine whether their TCRs bind to the same or different residues, we synthesized a panel of naturally occurring peptides and compared their ability to trigger the A9.2 clone and hybridomas. In addition to hydroxylation followed by glycosylation of Lys^264^, the CII256–270 peptide may undergo hydroxylation of Pro^258^; we therefore focused on peptides accordingly modified at those positions. Albeit with varying magnitude, the response patterns to synthetic peptides used were identical, regardless of the T cells stimulated (Figure 2a). Indeed, the presence of sugar moiety (Gal-Hyl^264^) was mandatory for T-cell activation, whereas hydroxylation of Pro^258 ^did not influence the recognition by any of the four TCRs. Because the heterologous CII256–270 sequence differs from that of mouse by a single conservative Glu^266^→Asp substitution, we also tested the ability of mouse Gal-Hyl^264 ^peptide to trigger T-cell reactivity. Notably, all clones were stimulated by the mouse glycopeptide, although at higher concentrations than bovine glycopeptide (Figure 2a). Heterologous Gal-Hyl^264 ^peptides, irrespective of Pro^258 ^hydroxylation, exhibited dose-dependent production of IL-2 by hybridomas with a threshold as low as 1 to 3 μmol/l as reaching a plateau at from 6 to 12 μmol/l. On the other hand, in the presence of mouse Gal-Hyl^264 ^the stimulating intensity varied between T-cell hybridomas and doses of at least 25 μmol/l were required to induce detectable responses (not shown).

### Integrity of galactose moiety but not its stereochemical position is an absolute requirement for T-cell activation

To unravel the molecular and structural basis for recognition of the CII256–270 glycopeptide by the TCRs, we synthesized chemically modified analogues and subsequently tested their ability to trigger the different T cells. As a first step, we explored the impact of alterations targeting the carbohydrate molecule. Protection of all of the hydroxy groups exposed on the galactose molecule (peptide GalPiv-Hyl^264^) resulted in complete loss of T-cell reactivity, whichever T cells were tested (Figure 2b). More precisely, replacement of galactose carried by Gal-Hyl^264 ^peptide with glucose (peptide Glc-Hyl^264^; specifically, substituting the axial HO-4 of galactose by an equatorial hydroxy group; Figure 1) totally elimiated the responses of A2G10 and A9E5 hybridomas but retained stimulation of A8E2 hybridoma. These findings point to the galactose HO-4 group as a key contact with the TCRs.

Further definition of the interactioins between galactose and TCRs was investigated by means of Gal-Hyl^264 ^derivatives modified at sugar anchor position (C-5) on hydroxylysine. Thus, two peptides were prepared: one with altered sugar orientation (peptide Gal [5*S*]-Hyl^264^) and the other with an additional methylated substitution at C5 (peptide Gal [5Me]-Hyl^264^). Compared with positive control stimulation obtained with the cognate peptide Gal-Hyl^264^, inversion of the configuration in peptide Gal [5*S*]-Hyl^264 ^markedly inhibited IL-2 production by A2G10 and A9E5 hybridomas. Indeed, much higher concentrations of the analogue were required for cell stimulation, and even at 100 μmol/l the magnitude of the response was half that elicited by Gal-Hyl^264 ^(Figure 2c). In contrast, the change in sugar orientation obtained in peptide Gal [5*S*]-Hyl^264 ^had little impact on recognition by A8E2 hybridoma and A9.2 clone. Regarding the steric hindrance created in the vicinity of the galactosyl moiety (peptide Gal [5Me]-Hyl^264^), this only moderately influenced activation of all the T cells tested (Figure 2c).

### The ε-primary amino group of Hyl^264 ^is a critical TCR-peptide contact in Gal-Hyl^264 ^specific recognition

**Figure 3:**
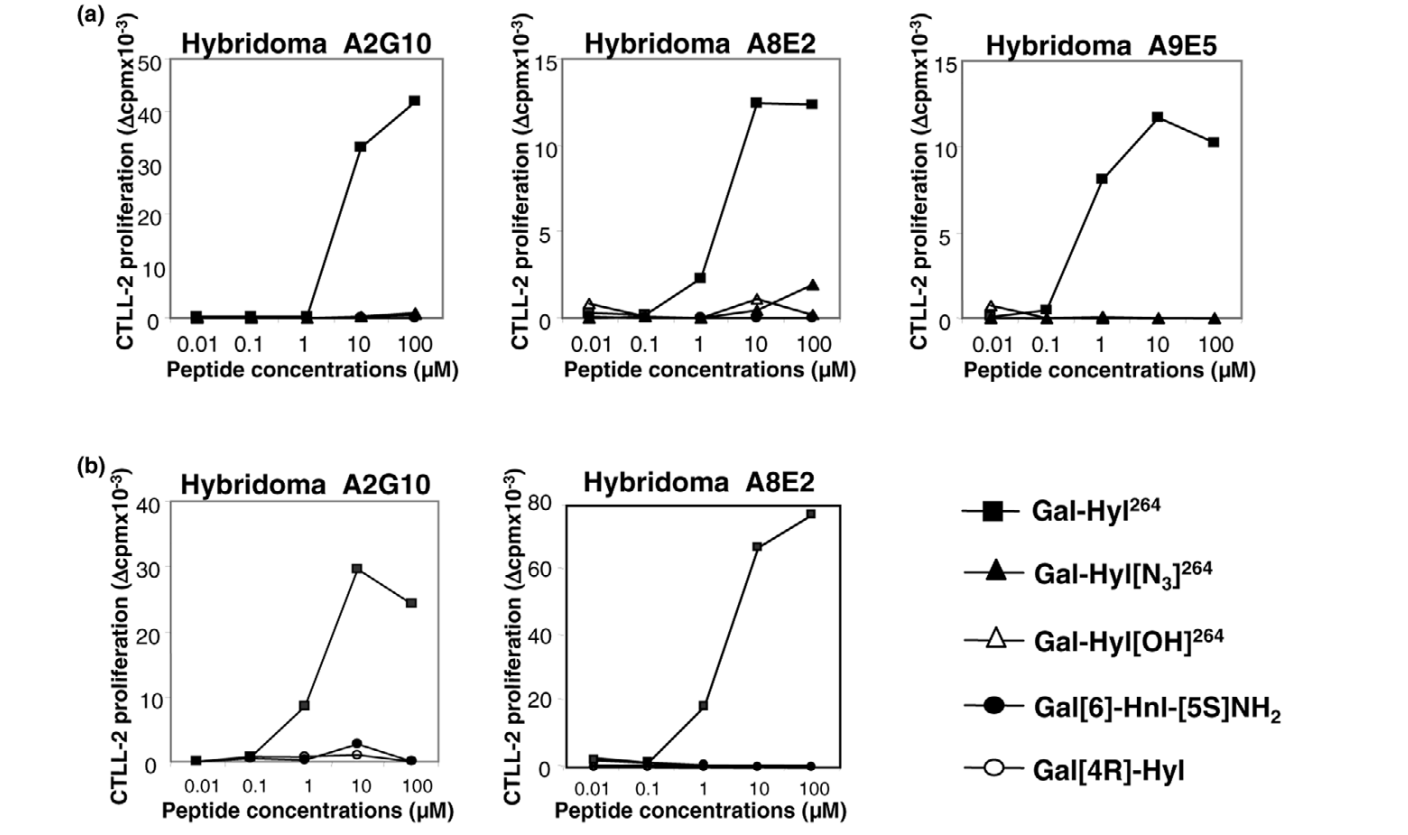
Responses of anti-CII T hybridomas upon stimulation with chemically modified CII256–270 glycopeptides. Data are expressed as means of two to four individual experiments. Blocking effects of alterations **(a) **reaching the ε-primary amino group of Hyl^264 ^or **(b) **strongly affecting the stereochemical position of sugar moiety. CII, collagen type II; CII256–270, immunodominant epitope of bovine type II collagen.

The next question we addressed concerned the role played by the hydroxylysine side chain of Gal-Hyl^264 ^epitope in peptide-TCR interaction. For this purpose, T cells were checked for their reactivity to synthetic peptides in which the ε-primary amino group of Hyl^264 ^was replaced by either an azido group (peptide Gal-Hyl [N_3_]^264^) or a hydroxy function (peptide Gal-Hyl [OH]^264^). In both cases, all of the T-cell responses were eliminated (Figure 3a). Similarly, the galactosylated non-natural amino acid hydroxynorvalin (Gal-Hnv) peptide, which lacks aminomethylene group of hydroxylysine, failed to stimulate A8E2 hybridoma and A9.2 clone (not shown).

### The relative position of the elements within Gal-Hyl^264 ^interacting with the TCRs is essential for T-cell activation

Having established that both galactose HO-4 and hydroxylysine side chain primary amino groups were key elements in the interaction of Gal-Hyl^264 ^peptide with the TCRs, we next focused on the importance of their relative spatial configuration for TCR triggering. Thus, two synthetic glycopeptides were prepared; the first one comprised a permutation of the carbohydrate and the amino groups (peptide Gal[6]-Hnl-[5*S*]NH_2_) and, in the second one, the anchor of galactose molecule on hydroxylysine was located at position C4 instead of C5 (peptide Gal [4R]-Hyl). Both peptides were then tested for their ability to elicit IL-2 production by A2G10 and A8E2 cells. Figure 3b shows that although the cognate glycopeptide Gal-Hyl^264 ^induced strong, dose-dependent responses, neither of the structural alterations totally abrogated the T-cell reactivity.

### Inhibition studies and immunogenicity of synthetic analogues

**Figure 4:**
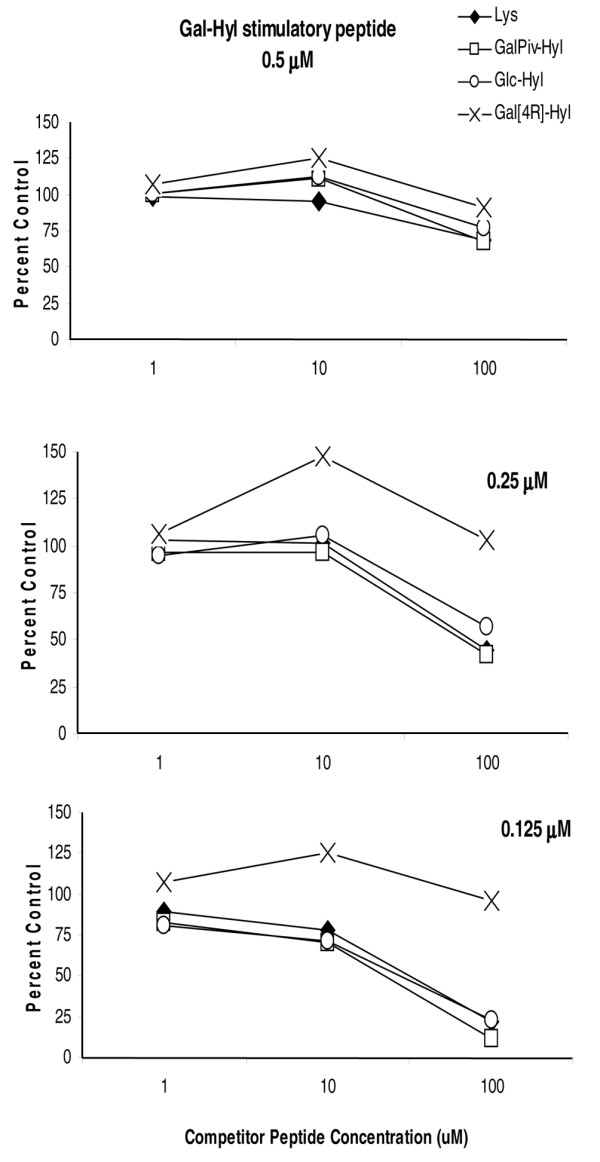
The inhibition of the response of the A8E2 hybridoma. Gal-Hyl peptide was used as the indicator peptide and various concentrations of competitor peptides were added to each assay. Data are expressed as the percentage of response in the absence of competitor and are representative of at least two separate experiments. The same results were obtained with A2G10 hybridoma.

Binding of the immunodominant glycopeptide CII256–270 to I-A^q ^molecule was assigned to two residues, namely Ile^260 ^and Phe^263^, and it was shown that glycosylation at position 264 did not change the MHC anchor positions [21]. Although none of the synthetic peptides used in this study were substituted at MHC binding positions, we explored whether the analogue glycopeptides were able to elicit an MHC restricted immune T-cell response. First, we pre-incubated synthetic analogues with APCs 2 hours before the addition of stimulatory glycopeptide and responsive hybridomas. The pre-incubation of APCs with two peptides modified at the sugar moiety (GalPiv-Hyl and Glc-Hyl) and the peptide without post-translation modification on the Lys^264 ^induced a dose-dependent inhibition of A8E2 hybridoma stimulation with the immunodominant glycopeptide Gal-Hyl^264^. Conversely, the Gal [4R]-Hyl elicited a moderate effect, probably because of the lesser avidity of this peptide with the MHC molecule (Figure 4). Similar results were observed with the two other hybridomas, A9E5 and A2G10 (data not shown).

**Figure 5:**
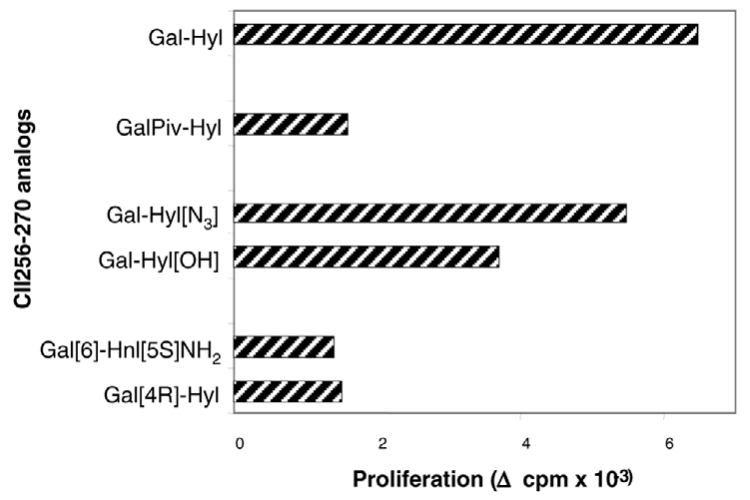
Immunogenicity of nonstimulating analogues. DBA/1 mice were immunized with glycopeptides in complete Freund's adjuvant as indicated, and their lymph node cells were tested 11 days later for their ability to proliferate *in vitro *in response to the immunizing peptide. Data are expressed as means of two to five mice per group. cpm, counts/min.

Second, we have explored whether the nonstimulating analogues were able to elicit a I-A^q ^restricted immune response by testing lymph node T-cell proliferation against the peptides in DBA/1 mice immunized with the respective peptides in CFA. Compared with the cognate epitope (Gal-Hyl^264^), the two glycopeptides modified at the ε-primary amino group of Hyl^264 ^(Gal-Hyl [N_3_] or Gal-Hyl [OH]) elicited substancial responses, whereas the pivoylated and both analogues altering the relative position of the elements (Gal[6]-Hnl-[5*S*]NH_2 _and Gal [4R]-Hyl) were less immunogenic (Figure 5).

These findings confirm that modified glycopeptides were able to generate a T-cell response and to bind the MHC molecule present at the surface of APCs.

## Discussion

This paper focuses on the molecular characterization and spatial configuration involved in the recognition of the galactose moiety within the CII256–270 immunodominant epitope. This was achieved using several closely related T-cell clones and hybridomas specific exclusively for the galactosylated form of the peptide. We identified two contact points to be critical for TCR triggering and identified potential constraints on the binding orientation.

In the present study, we probed the fine specificity of four CII-specific T-cell clones that carry TCR expressing a unique rearranged α chain (Vα17/Jα20) associated with β chains using Vβ1, Vβ4, or Vβ10 gene segments but sharing almost identical βCDR3 sequences [14]. All of these T cells strictly recognized the carbohydrate moiety linked to Hyl^264 ^within the CII256–270 epitope because they were activated neither by the unglycosylated peptide nor by the peptide carrying a fully protected galactose molecule. Furthermore, the CII256–270 epitope most often undergoes hydroxylation of the Pro^258 ^residue, but such modification had no influence on sugar-mediated TCR triggering in any T cells tested. Interestingly, the T cells raised in mice immunized with bovine CII cross-reacted with mouse galactosylated peptide (which only differs by a Glu^266^→Asp substitution). The fact that the magnitude of the response to self peptide was lower and positive stimulation required higher concentrations than with heterologous peptide is unlikely to be due to greater steric hindrance of Asp versus Glu^266^, because the former residue has a shorter side chain. Alternatively, the difference may rely on the poor affinity to MHC of mouse peptide compared with heterologous peptide [22]. It is noteworthy that, in various situations involving autoimmunity, pathogenic T cells were shown to react to self peptides with low affinity for MHC class II molecules, indicating that such cells escape tolerance induction and cause autoimmunity [23,24]. Because homologous CII is known to induce chronic arthritis in DBA/1 mice [25], the glycopeptide-specific autoreactive cells may play a central role in perpetuating inflammation and joint destruction during the course of CIA.

The occurrence of glycopeptide reactive T cells has been documented in numerous systems, including CD4^+ ^and CD8^+ ^T-cell subsets. A previous study that analyzed the TCR repertoires used for recognition of CII(256–270) epitope according to its potential post-translational modifications at position 264 [12] concluded that the Gal-Hyl^264 ^glycopeptide is immunodominant; specifically, this glycopeptide stimulated most of the CII-specific T cells, among which one hybridoma – generated from immunized DBA/1 mice – had a TCR structure very similar to that of the A9.2 clone. The present work supports this conclusion and extends it to other TCRs. All of the hybridomas and T cells we used in the study exhibited the same recognition profile, although the intensity of the responses differed according to the hybridoma concerned. This observation is possibly attributable to pinpoint differences in TCR structure. Notably, within the CDR3β, only one D-region nucleotide varies in either T-cell clone or hybridomas, resulting in expression of four different amino acids at this position [14]. This D-region encoded residue may thus directly come into contact with the anchored sugar part of the peptide and affect the level of T-cell responses, as shown in Figure 2.

Using a large panel of synthetic structural analogues of the natural epitope recognized by the T cells, we were able to define two critical molecular contacts of Gal-Hyl^264 ^interacting with the TCR and to identify a certain TCR flexibility in this recognition process. One of the key elements in the peptide-TCR interaction is the HO-4 group of the galactosyl moiety, because the substitution of galactose by glucose, which only affects the inversion of the stereochemistry of hydroxy group at position C4, eliminated off T-cell activation. In addition, it was reported that removal of any of the other hydroxy groups did not alter the responses of Gal-Hyl^264^-specific hybridomas [26]. The second molecular contact within the glycosylated peptide that is not dispensable for TCR triggering is the side chain primary amino group of Hyl^264^, because analogues chemically modified at that position were barely recognized by the T cells. It is plausible that the primary amine at ε position participates in electrostatic interactions with negatively charged residues of the TCR. Alternatively, the ε-amino group can help to render the galactose spatial configuration suitable for TCR recognition by bridging to Glu^266 ^side chain, conferring higher stability upon the galactosyl moiety. The fact that the peptide synthesized with altered sugar orientation (Gal [5*S*]-Hyl peptide) activated T cells to a lesser degree favours such a hypothesis.

A9E5 and A8E2 T cell hybridomas differed in the TCR sequences by only one amino acid (Ala versus Val, respectively) in the CDR3 region of the TRB chain [15], and A8E2 but not A9E5 responded to Gal [5*S*]-Hyl peptide stimulation. Interestingly, the relative position of the two key elements within the cognate peptide for TCR stimulation is of crucial importance. Slight changes, such as the introduction of a methylene group attached to carbon C5, only minimally influenced the levels of T-cell responses, pointing to a certain degree of TCR flexibility. In contrast, the drastic stereochemical modifications caused by permutation of sugar and NH_2 _at the C5 position or by a shift of galactose anchor from C5 to C4 were detrimental to TCR engagement. It would be of interest now to test whether the modifications of the immunodominant CII epitope described herein could induce particular T-cell cytokine production patterns and whether the different modified peptides could have a protective/aggravating effect *in vivo*.

## Conclusion

Collectively, our findings provide strong new experimental evidence that integrity of both galactose HO-4 and hydroxylysine side chain primary amino groups are mandatory for TCR activation. Thus, TCR interactions with peptide-MHC are topologically constrained, although some conformational flexibility can occur at the binding interface. Identification of Ileu^260 ^and Phe^263 ^as anchors in the P1 and P4 pockets of A^q^, respectively, has been documented in different studies, thereby providing experimental support for molecular modelling of the complex between A^q ^molecule and CII256–270 peptide [21,22,26]. Because the αβ TCRs were reported to dock onto the peptide-MHC with the Vα domain of the TCR positioned over the amino-terminal half of the peptide and the Vβ domain over the carboxyl-terminus, it is plausible that the P5-Gal-Hyl^264 ^residue is facing the CDR3 α and β loops located in the centre of the TCR, allowing direct pinpoint contact between the HO-4 position of carbohydrate and TCR. In accordance with this hypothesis, recent work focusing on the crystal structure of an autoimmune TCR complexed with class II peptide-MHC involved in murine experimental allergic encephalomyelitis [27] revealed that there were few specific contacts between the TCR CDR3 loops and the cognate peptide.

## Abbreviations

APC = antigen-presenting cell; CFA = complete Freund's adjuvant; CIA = collagen-induced arthritis; CII = type II collagen; CII256–270 = immunodominant epitope of bovine CII; IL = interleukin; MHC = major histocompatibility complex; RA = rheumatoid arthritis; TCR = T-cell receptor.

## Competing interests

The authors declare that they have no competing interests.

## Authors' contributions

SG was responsible, along with MAB, for the execution of most of the experiments as well as drafting the manuscript. MAB was responsible for the execution of most of the experiments. JM performed the majority of the studies regarding peptide synthesis and purification. SM was responsible for the execution of all proliferative experiments. JPB gave valuable assistance during the period of experimentation and manuscript preparation. GG gave valuable assistance during the period of experimentation, particularly for peptide synthesis and purification, and manuscript preparation. CF was responsible for most of the data analysis; she was responsible for study design coordination and the writing of the manuscript, and interpretation and discussion of the data. GC was responsible for most of the data analysis; he was responsible for study design coordination and writing of the manuscript, and also interpretation and discussion of the data.
